# Cognitive Impairment Without Dementia: A Treatable Case of Dural Arteriovenous Fistula

**DOI:** 10.7759/cureus.87866

**Published:** 2025-07-13

**Authors:** Kamand Khalaj, Rima El Atrache, Anit K Behera, Irma Duncan, Thien Thanh Nguyen, Kasra Rahbar

**Affiliations:** 1 Radiology, University of Texas Health Science Center at Houston, Houston, USA; 2 Neurology, Baylor College of Medicine, Houston, USA; 3 Vascular Neurology, Advocate Lutheran General Hospital, Park Ridge, USA; 4 Neurology, Michael E. DeBakey Veterans Affairs Medical Center, Houston, USA; 5 Interventional Radiology, Michael E. DeBakey Veterans Affairs Medical Center, Houston, USA

**Keywords:** cognitive decline, dementia, dural arteriovenous fistula, embolization, mild cognitive impairment, onyx

## Abstract

Dural arteriovenous fistula (DAVF) can present with a wide range of symptoms depending on its location and the degree of arteriovenous shunting. Rarely, they may manifest as cognitive impairment due to chronic venous dysfunction. This is the case of an elderly man evaluated for subacute onset of memory loss without dementia. Magnetic resonance imaging (MRI) of the brain revealed a subtle subarachnoid vascularity in the right occipital lobe, without cerebral edema or parenchymal abnormality. Digital subtraction angiography (DSA) confirmed a low-flow Borden type III DAVF, draining via an occipital cortical vein into the basal vein of Rosenthal and the vein of Galen. Subjective cognitive symptoms resolved following successful treatment with transarterial Onyx embolization. The role of DAVFs in mild cognitive impairment without dementia is likely underrecognized due to their rarity and inherent difficulties in diagnosis. This suggests a potential role for detailed cognitive evaluation in guiding the management of DAVFs.

## Introduction

Intracranial dural arteriovenous fistulas (DAVFs) are an uncommon abnormal connection between one or more arteries and veins (bypassing the capillaries, thereby resulting in a shunt) within the brain dura mater layer. DAVFs are believed to be acquired from neoangiogenesis and are triggered by various causes, including venous thrombosis, trauma, or craniotomy [1]. In many cases, no definite cause is identified, in which case it is assumed that the underlying event leading to the DAVF is remote or unrecognized. DAVFs are distinct in their pathogenesis, natural history, and management from cerebral arteriovenous malformations (AVMs). Unlike DAVFs, cerebral AVMs are defined by arteriovenous shunting within an intraparenchymal nidus and are generally thought to be developmental rather than acquired.

DAVFs can present with a wide range of clinical symptoms. The clinical presentation depends on the anatomical location of the fistula and the severity of arteriovenous shunting [2]. Common symptoms and signs, including headache, pulsatile tinnitus, bruit, and ophthalmological symptoms, are variably present. Some cases are asymptomatic or subclinical, either discovered incidentally or remaining clinically silent until the initial presentation with acute hemorrhage. Asymptomatic DAVF is incidentally detected at a low rate on imaging, but the true incidence is unknown and likely underestimated due to the inherent limitations of noninvasive imaging [3].

Rarely, DAVFs can present primarily with cognitive impairment or dementia [4]. The clinical presentation may be distinct based on the venous drainage pattern. Superficial venous drainage may be associated with dysfunction of specific cortical areas, for example, impaired executive function, personality changes, or aphasia. Deep venous drainage may be associated with tremors and bradykinesia from thalamic involvement. Additional neurological deficits, including motor weakness, sensory changes, and gait abnormalities, may be present.

This case report presents an elderly man evaluated for the subacute onset of memory loss. Magnetic resonance imaging (MRI) revealed subtle subarachnoid vascularity in the right occipital lobe. This was confirmed with digital subtraction angiography (DSA) to be a low-flow Borden type III DAVF, draining via a right occipital cortical vein into the basal vein of Rosenthal and the vein of Galen. Successful transarterial embolization with complete occlusion was followed by the resolution of cognitive symptoms.

## Case presentation

A 73-year-old man with a past medical history significant for essential tremor and a remote history of head trauma with no reported history of psychiatric conditions or mood disorders presented with a chief complaint of cognitive symptoms that developed over several months. He reported memory lapses, disorientation, and poor concentration. At baseline, he was functionally independent and both intellectually and physically active. Multiple episodes of an acute confusional state were especially alarming for the patient and his family, which prompted them to seek medical attention. He denied experiencing headaches or pulsatile tinnitus. Neurological exam revealed no abnormalities in orientation, language, attention, repetition, comprehension, naming, and memory recall; cranial nerves 2-12 were intact; right upper and lower extremities and left upper and lower extremities were 5/5 strength in all muscle groups; sensation was intact to light touch and pinprick; cerebellar function was intact to finger-nose-finger and heel-to-shin; and gait was normal. There was a low clinical suspicion for seizures; therefore, an electroencephalogram was not done.

**Figure 1:**
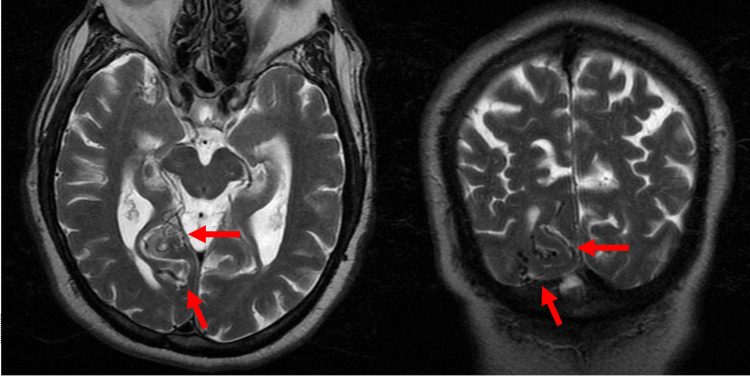
T2-weighted MRI The (a) axial and (b) coronal planes demonstrate subtle subarachnoid serpiginous vascular flow voids (arrows)

MRI of the brain without contrast was performed for work-up of cognitive impairment. T2-weighted imaging demonstrated subtle subarachnoid serpiginous vascular flow voids in the right occipital lobe (Figure 1).

Initially, this vascularity was suspected to be an incidental AVM. However, the confinement of the vascularity to the subarachnoid space without an intraparenchymal nidus was inconsistent with AVM. The subarachnoid vascularity represented mildly ectatic cortical draining veins of a DAVF. Of significance, there was no evidence of cerebral edema or parenchymal abnormality, and MRI was otherwise negative for an alternative explanation for the cognitive symptoms. 

**Figure 2:**
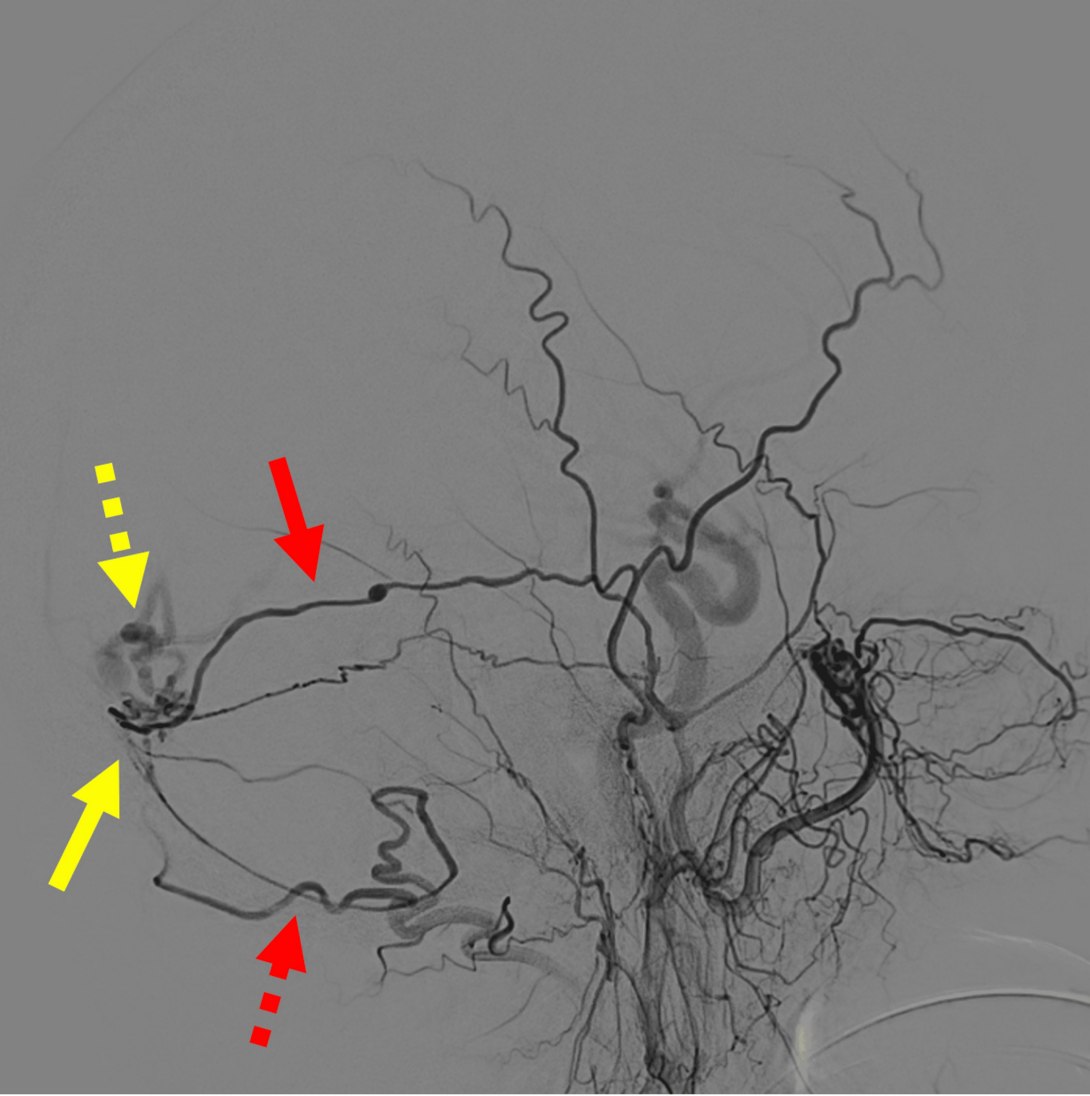
Selective catheter angiography Selective catheter angiography performed in the right external carotid artery demonstrates the location of the fistula with neoangiogenesis in the wall of the transverse sinus (solid yellow arrow). Arterial supply comes from the middle meningeal artery (solid red arrow), which has a small flow-related aneurysm, and the occipital artery (dashed red arrow). Low-flow arteriovenous shunting with early venous opacification of a mildly ectatic, tortuous occipital cortical draining vein (dashed yellow arrow)

**Figure 3:**
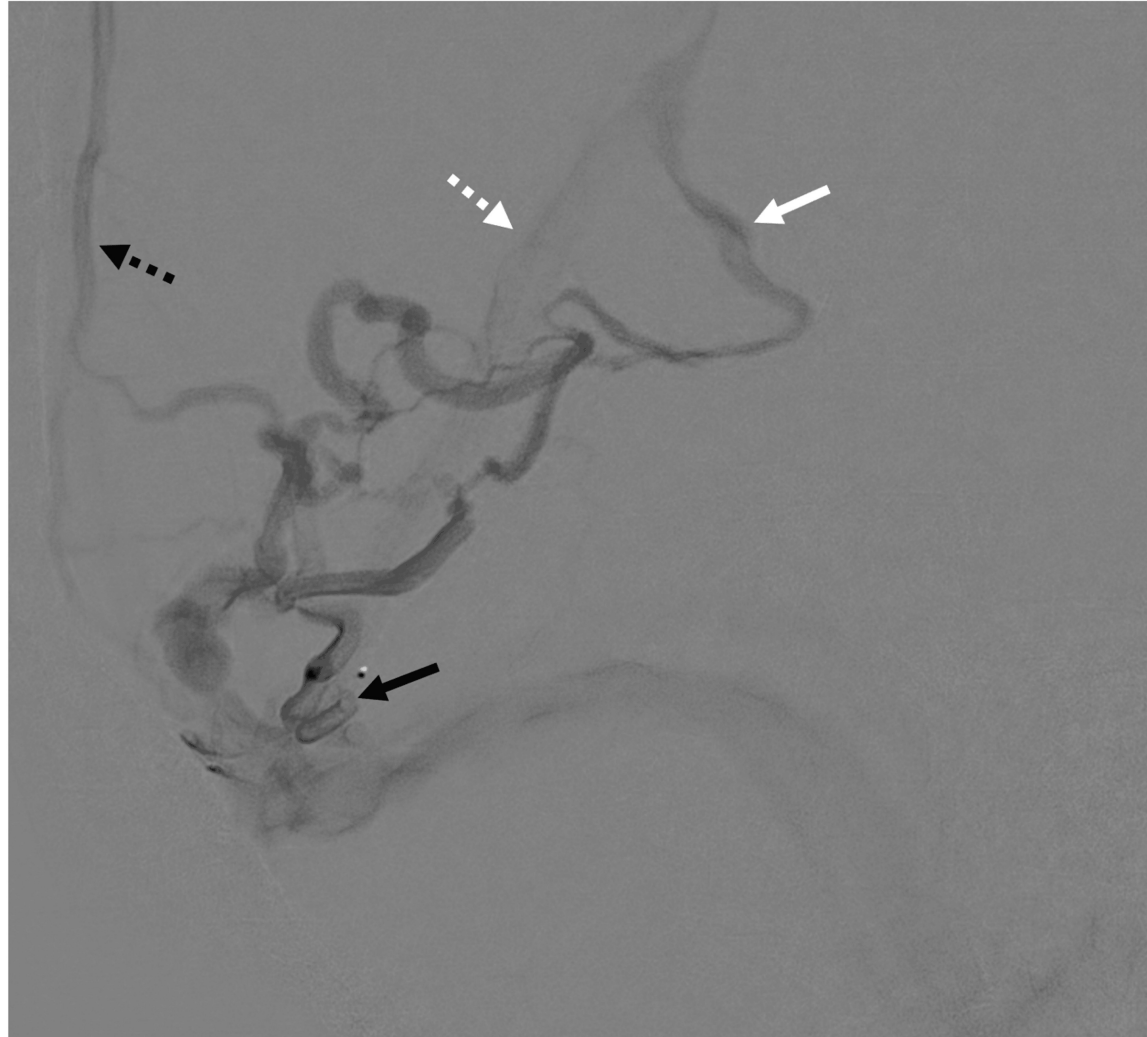
Superselective microcatheter angiography Superselective microcatheter angiography of the right middle meningeal artery feeder in the delayed phase demonstrates the anatomy of the draining vein. The occipital cortical draining vein originates from the wall of the transverse sinus (solid black arrow) and drains through a network of ectatic, tortuous cortical veins into the basal vein of Rosenthal (solid white arrow), subsequently into the vein of Galen and straight sinus (dashed white arrow). There is a separate small vein draining into the superior sagittal sinus (dashed black arrow)

DSA was performed and confirmed a low-flow Borden type III DAVF. The angioarchitecture was characterized by a single-hole fistula located within the wall of the right medial transverse sinus, supplied by branches of the right middle meningeal artery and occipital artery, and draining via a single occipital cortical vein primarily into the right basal vein of Rosenthal and vein of Galen (Figures 2, 3). 

**Figure 4:**
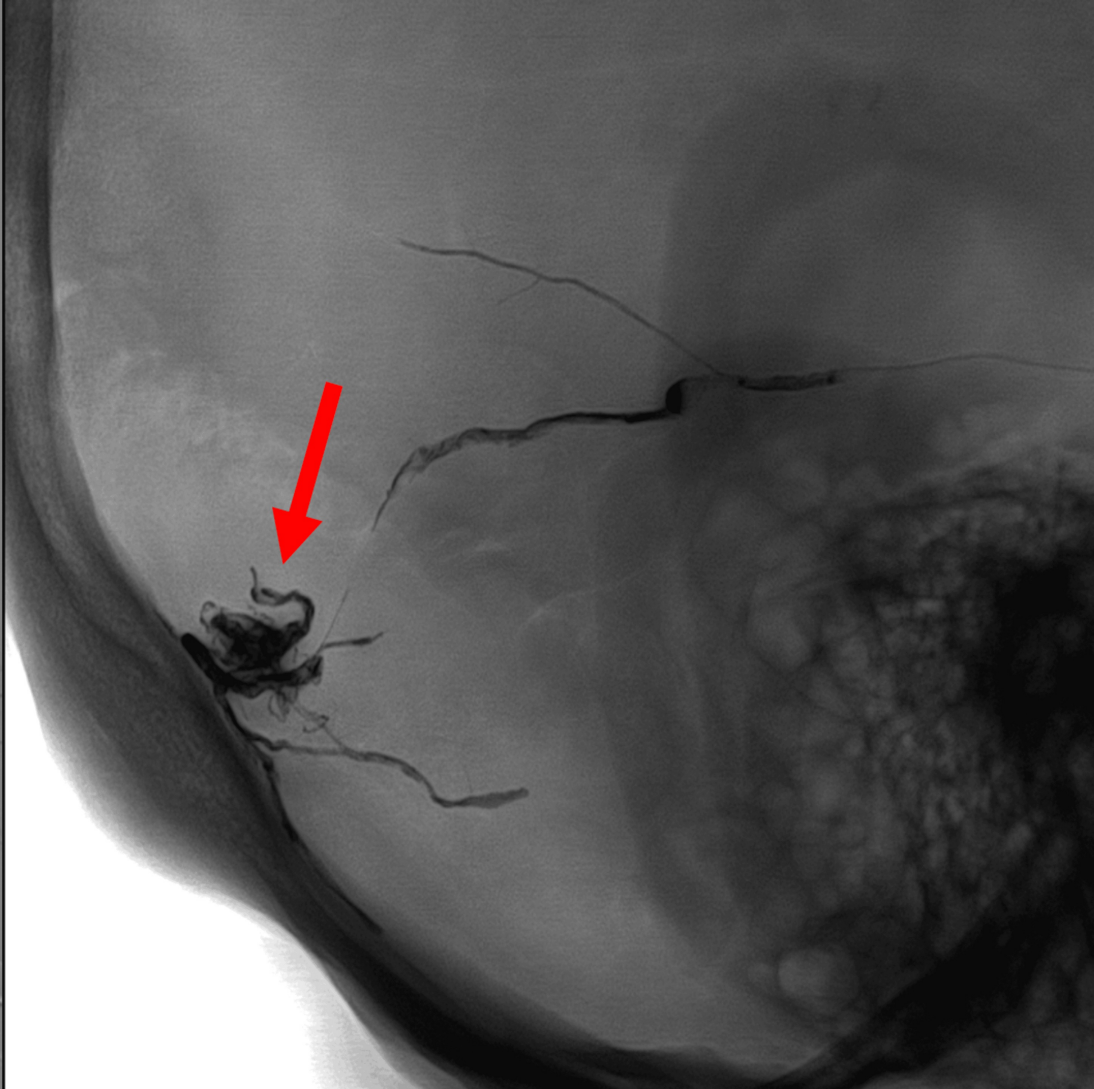
Post-embolization image of the Onyx cast demonstrates occlusion of the fistulous point with penetration into the draining vein (arrow)

Treatment with transarterial embolization was performed, given the hemorrhagic potential in the presence of CVD. The patient underwent successful embolization with distal microcatheterization of the right middle meningeal artery for Onyx injection, achieving penetration into the draining cortical vein, thereby ensuring complete occlusion of the fistula (Figure 4). 

There was no evidence of residual arteriovenous shunting on post-embolization angiography. The patient experienced transient visual auras and headaches in the first few hours following embolization, which resolved without complication. Six months later, the follow-up angiogram revealed durable complete occlusion of the DAVF without recurrence. 

Two weeks following embolization, the patient endorsed the resolution of cognitive symptoms. In his own words, he states, “I’ve regained my clarity.” Comprehensive neuropsychological testing performed at five months post-embolization was broadly normal. On clinical follow-up one year later, the improvement in cognitive symptoms was sustained. 

## Discussion

This case report describes the diagnosis and treatment of a low-flow Borden type III DAVF in the setting of cognitive impairment without dementia. Successful treatment was followed by the subjective resolution of memory loss. While the degree of CVD was not severe enough to cause edema or parenchymal abnormality on MRI, the patient's cognitive symptoms may be explained by venous drainage via the basal vein of Rosenthal and the vein of Galen, causing venous stasis in the hippocampal veins [5] or thalamic veins [6].

Aggressive manifestations of DAVFs are generally a consequence of venous hypertension and include intracerebral hemorrhage, nonhemorrhagic neurological deficits, and seizures due to cortical irritability. The Borden classification [7] and Cognard classification [8] systems are commonly used to predict the risk of hemorrhage based on the pattern of venous drainage. The presence of cerebral venous drainage (CVD) is the single most important angiographic characteristic in predicting the risk of aggressive behavior. CVD can be due to direct fistulous drainage into a cortical vein (Borden type III and Cognard types III and IV) or indirect reflux into a cortical vein from a venous sinus (Borden type II and Cognard types IIb and IIa+b). Zipfel et al. modified the Borden classification to include whether CVD is causing symptoms (excluding pulsatile tinnitus or ophthalmologic symptoms) in evaluating the hemorrhagic risk [9]. The annual rate of hemorrhage for asymptomatic CVD is estimated to be 1.4-1.5%, while symptomatic CVD is estimated to be 7.4-7.6%. Borden type I DAVFs without CVD are usually associated with a benign natural history and a 2% risk of developing CVD on follow-up [10]. The presence of CVD usually warrants treatment, while DAVFs without CVD can be safely monitored unless symptoms require treatment.

MRI is usually the preferred modality for DAVF diagnosis, given better conspicuity of findings compared to CT. The presence of cerebral edema is a red flag and corresponds to CVD in the affected venous territory. In severe cases, cerebral edema can be associated with ischemia or hemorrhage. Hypervascularity from the DAVF itself can be subtle or inapparent on MRI or computed tomography (CT). Specific MRI techniques may improve detection of arteriovenous shunting or venous reflux, including gadolinium-enhanced T1-weighted imaging [11], susceptibility-weighted imaging [12], time-of-flight magnetic resonance angiography (MRA) [13], and time-resolved contrast-enhanced MRA [14]. Although these techniques can improve accuracy, negative noninvasive imaging does not rule out the diagnosis of DAVF, and DSA remains the definitive test and gold standard.

Numerous reports have described DAVFs as a rare, reversible cause of severe, progressive dementia [4]. The Neuropsychology in dural ArterIal Fistula (NAIF) study is a prospective study of 32 patients who underwent embolization for DAVF, demonstrating objective improvement in multiple domains of cognitive testing at three-month follow-up compared to pretreatment baseline [15]. Interestingly, cognitive improvement was independent of Cognard classification. This suggests that DAVFs, which may otherwise remain untreated due to their low hemorrhagic risk, could have a cognitive benefit from embolization. However, future studies are needed to confirm this benefit. A retrospective multicenter analysis of the Consortium for Dural Arteriovenous Fistula Outcomes Research (CONDOR) Consortium, consisting of 1077 cases of DAVF, compared 60 patients with cognitive impairment to 60 control patients [16]. The presence of cognitive impairment was associated with venous hypertension in all cases and was also significantly associated with venous sinus stenosis, a higher number of arterial feeders and draining veins, and venous ectasia. A trend toward achieving asymptomatic status was observed following successful treatment. Finally, there are many case reports (this current case report adds to this body of literature) that showed a dramatic improvement or reversal of cognitive symptoms following successful treatment. But the limitations with these case reports include the lack of rigorous control groups, and the diagnosis of dementia was made based on clinical judgment, and formal neuropsychological testing was either not performed or not reported [17].

However, the role of DAVFs in milder degrees of cognitive impairment without dementia is likely under-recognized, due to inherent challenges in clinical and imaging diagnosis. Milder cognitive symptoms may be associated with low-flow arteriovenous shunting and absence of cerebral edema or parenchymal abnormalities on MRI, making the imaging diagnosis more difficult. Clinical assessment of cognitive function relies to an extent on the patient and family’s subjective narrative of cognitive decline relative to premorbid status. Neuropsychological testing is useful to objectively measure cognitive function, but it cannot be used in isolation. A comprehensive evaluation must exclude confounding neurological, medical, or psychiatric conditions. Despite the challenges in diagnosing and studying this entity, recent data is emerging that elucidates the relationship between DAFVs and cognitive impairment.

A limitation of this case report is the absence of baseline neuropsychological testing prior to treatment. Given the hemorrhagic risk, it was in the patient’s best interest to proceed with embolization without delay rather than await formal neuropsychological testing, which often can take several months to perform. Nevertheless, it is reasonable to conclude that the cognitive symptoms were in fact caused by the DAVF, given the resolution of symptoms following treatment, sustained improvement on follow-up, and the absence of alternative explanation or neurodegenerative process.

Resting state functional MRI has demonstrated alterations in functional connectivity that could be an imaging marker for DAVF with cognitive impairment [18,19]. These alterations were reversible following successful treatment [19]. We acknowledge that our study did not include advanced imaging techniques such as arterial spin labeling or resting-state fMRI, which may be more sensitive. This represents a limitation of our work and highlights the need for further studies employing these modalities to better support our hypothesis.

## Conclusions

DAVFs are a rare but potentially reversible cause of severe, progressive cognitive impairment. This case raises the possibility of an association between DAVFs and milder forms of cognitive impairment, although a causal relationship remains unproven. The diagnostic challenge is compounded by the frequent absence of cerebral edema or parenchymal abnormalities on conventional MRI, which may contribute to under-recognition in patients without overt dementia. Regardless, if findings are not correctly recognized, cognitive symptoms can be misattributed to other, more common neurological or psychiatric conditions. Since parenchymal findings on MRI can be nonspecific, work-up can sometimes be misdirected toward neoplastic or inflammatory causes, which is a known pitfall. Clinicians and radiologists must maintain a high index of suspicion and consider DSA when appropriate, as cognitive symptoms can be reversible with DAVF treatment.
